# Erosion of Cardiovascular Implantable Device: Conservative Therapy or Extraction?

**DOI:** 10.7759/cureus.12032

**Published:** 2020-12-11

**Authors:** Randa N Tabbah, Bernard Abi-Saleh

**Affiliations:** 1 Cardiology, Centre Hospitalier Universitaire Notre Dame de Secours, Beirut, LBN; 2 Cardiology, American University of Beirut Medical Center, Beirut, LBN

**Keywords:** pocket infection, erosion, extraction, conservative therapy, antibiotics

## Abstract

The standard of care for device infection is normally a complete removal of the implantable system, including lead extraction in local or systemic infection cases. Despite the importance of lead extraction techniques, these techniques are complex and have some major risks. Success rates were high, but they are less favorable in patients with several comorbidities.

An 80-year-old male presented for device erosion. The patient is known to have several cardiac comorbidities: a transcatheter aortic valve replacement (TAVR), mitral clips for severe aortic stenosis, mitral regurgitation, dual-chamber implantable cardioverter defibrillators (ICD) for secondary prevention. Several weeks ago, he noted tenderness and redness at the site of his device pocket, and his physician, after checking his wound, suggested a possible skin irritation with no systemic infection and started antibiotics treatment. Two weeks later, he noted thinning of the skin around the device with a hematoma and ecchymosis, and slight skin erosion. Strategies for assessment of the wound and pocket cleaning were taken. The strategy was to remove the left-sided device and keep the leads since the patient lately has no elevated inflammatory labs, negative cultures, no fever, nor signs of vegetation on transesophageal echocardiography (TEE) and refused any additional examination as positron emission tomography (PET) scan, and reimplant a new system on the contralateral side. The procedure was divided into two sequences: extracting the device and after one-week implantation of a right-sided new system. In this case, chronic antibiotics were discussable to decrease the recurrence rate, but they did increase the severity of the patient's thrombocytopenia.

Despite extraction being the gold standard of treatment in most cases of devices with local and systemic infection, there are some frail patients with several comorbidities where extraction is unbearable due to its major risks and complex procedure. In these specific cases with local infection and device erosion with no signs of any systemic infection, conservative therapy could be a viable option.

## Introduction

Cardiovascular implantable electronic device (CIED) infection is an important issue in this era due to increased device implantation, population growth, and adoption of the guidelines. Thus, an increase in infection might lead to long hospitalization, major comorbidities, and death. The standard of care for device infection is normally a complete removal of the implantable system, including lead extraction in case of local or systemic infection [1-5]. Local disinfection techniques were associated with a higher failure rate, morbidity, and mortality. Despite the importance of lead extraction techniques, these techniques are complex and have some major risks. Success rates were high, but they are less favorable in patients with several comorbidities [6-8]. We present a case of a frail patient with local pocket erosion treated with a successful conservative technique.

## Case presentation

**Figure 1:**
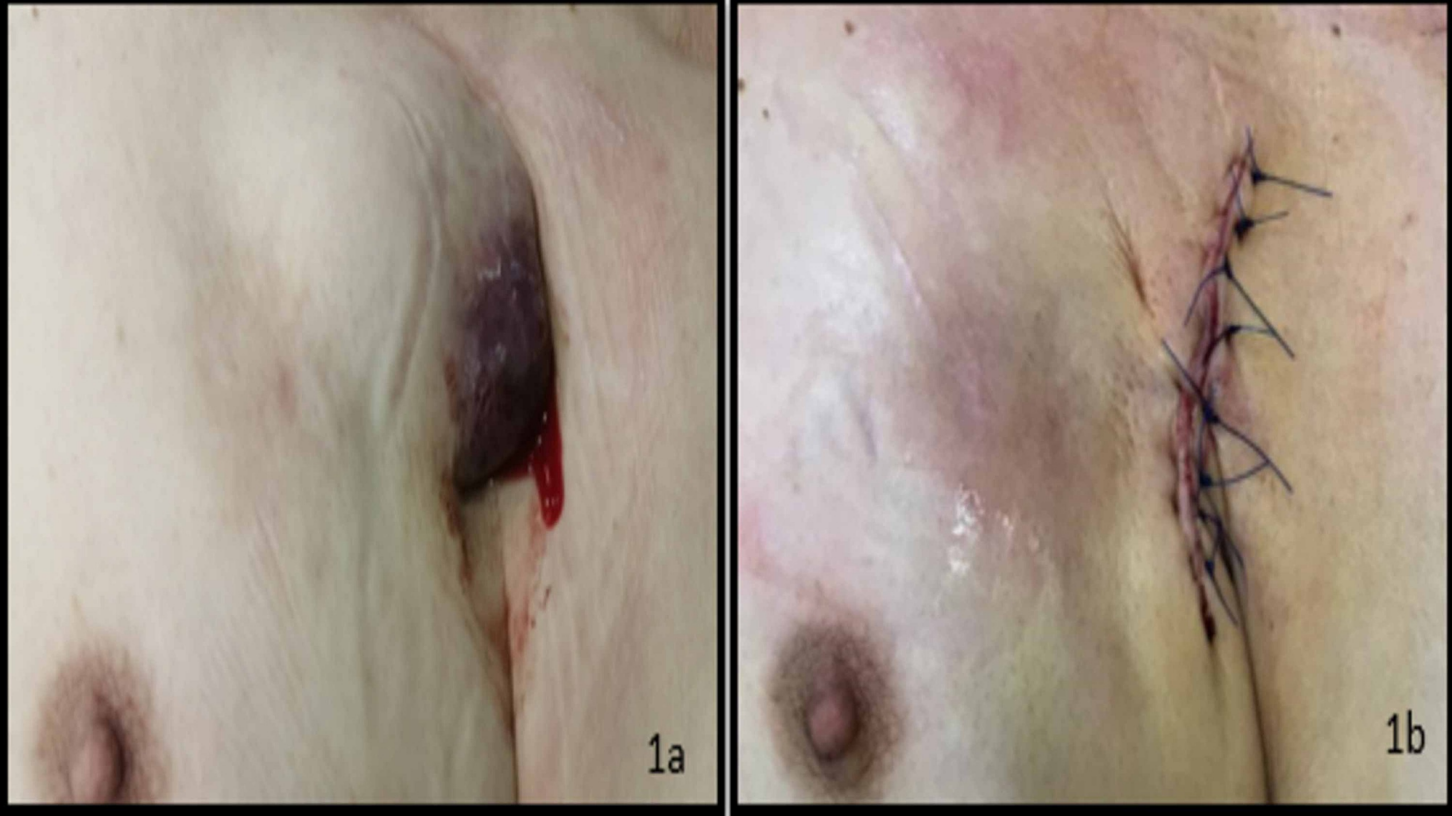
Before and after device explant 1a: device hematoma and erosion 1b: surgical debridement and explant of the device

An 80-year-old male presented with device erosion. The patient is known to have severe aortic stenosis with a severe mitral regurgitation treated with transcatheter aortic valve replacement and three mitral clips since he was at high risk for surgery, hypertension, diabetes, dyslipidemia, coronary artery disease, chronic kidney disease, chronic obstructive pulmonary disease, idiopathic thrombocytopenia and a dual-chamber implantable cardioverter-defibrillator (ICD) for secondary prevention and sick sinus syndrome eight years ago. Several weeks ago, he noted tenderness and redness at his device pocket site and consulted a cardiologist for a checkup. After checking his wound, his physician suggested a possible skin irritation and started him on antibiotics for further caution after echocardiography was done, and no signs of possible vegetations were noticed. Transesophageal echocardiography (TEE) was done weeks after and seemed to be negative for signs of endocarditis. Despite his wound tenderness, the patient didn’t have any fever or night sweats, nor fatigue. Two weeks after, he noted thinning of the skin around the device with a hematoma and ecchymosis (patient on anticoagulation for paroxysmal atrial fibrillation) and a slight skin erosion (Figure 1-a). No history of any specific trauma was noticed. His lab tests revealed normal white blood cell count, a CRP of 18 with a creatinine clearance of 50ml/min/1.73m2. Three times performed, hemocultures came negative. Cultures from the wound were also sterile. The patient refused any complex procedures and preferred a more conservative technique. 

Strategies for assessment of the wound and pocket cleaning were taken. The strategy was to remove the left-sided device and keep the leads since the patient lately has no elevated inflammatory labs, negative cultures, no fever nor signs of vegetation on TEE and refused any additional examination as positron emission tomography (PET) scan, and reimplant a new system on the contralateral side. The device was checked before the decision, and the patient seems to be nondependent with an acceptable underlying rhythm of 40 beats per minute (after beta-blockers and amiodarone were stopped). The procedure was divided into two sequences: extracting the device and after one-week implantation of a right-sided new system.

An incision was made in the infraclavicular area parallel to the middle third of the clavicle with sharp and blunt dissection, where the defibrillator generator was extracted. An extensive debridement was done. Fibrous and necrotic tissues were dissected as well as all the fatty tissues around them. No signs of pus and tissues were sent to pathology. Irrigation with the saline solution first, then iodine was performed followed by antibiotics. Capping and abandoning leads were completed. Two layers, one deep and another superficial was the strategy when suturing the wound. The patient was on intravenous teicoplanin and discharged home (Figure 1-b).

**Figure 2:**
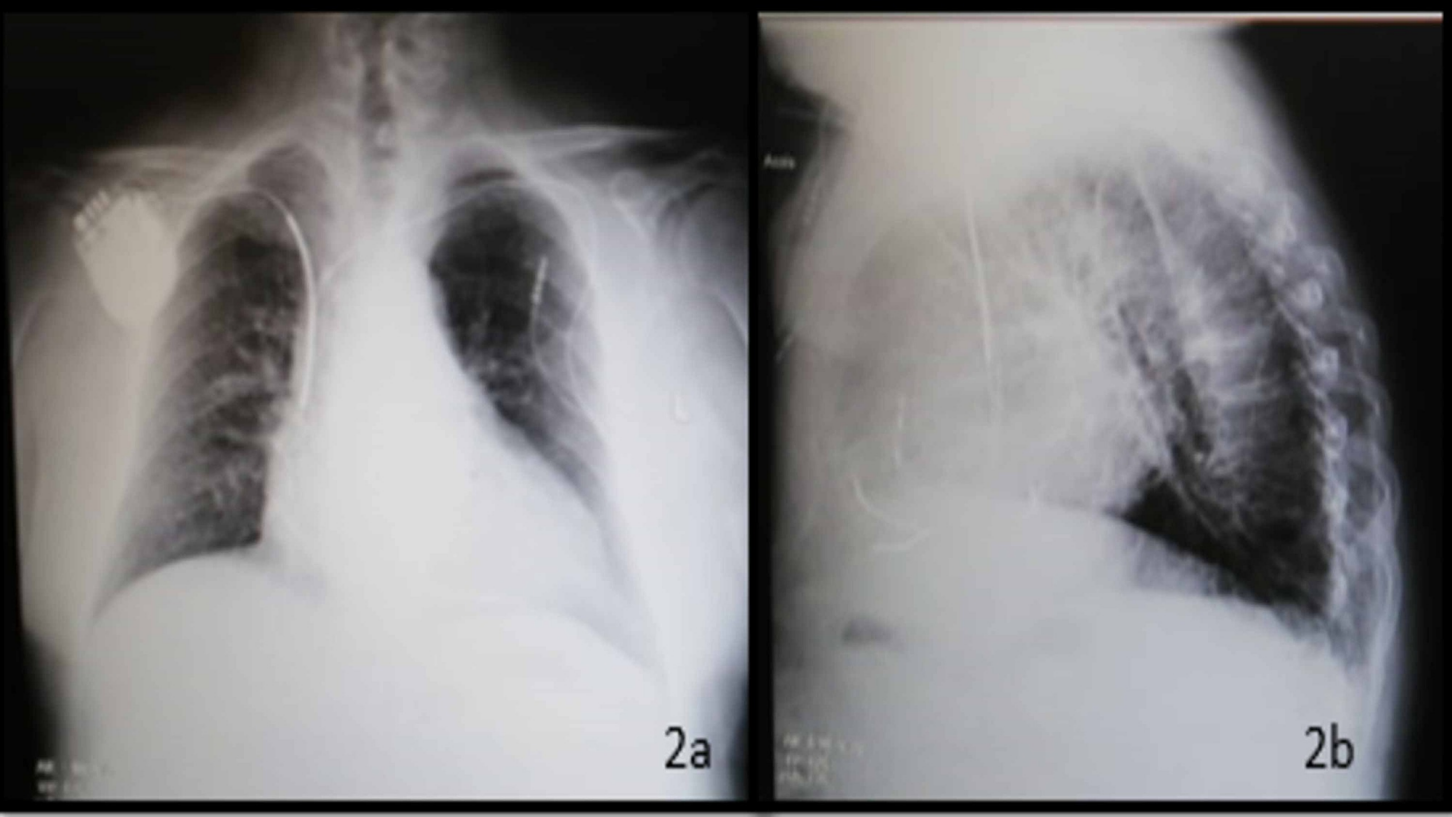
Device implantation on the right side 2a: chest X-ray posteroanterior (PA) view 2b: chest X-ray lateral view

After one week, the patient was readmitted, and the right-sided system was implanted. An incision was made in the infraclavicular area parallel to the middle third of the clavicle, and a subcutaneous pocket was created with sharp and blunt dissection where the defibrillator generator was implanted. Two subclavian access were made. The right ventricle and the right atrial leads were placed and yielded excellent thresholds. Irrigation with antibiotics was performed. Two layers of bioresorbable sutures (one deep and the other superficial) were used for pocket closure** **(Figure 2). During the hospitalization, the patient underwent an antibiotics side effect aggravating his thrombocytopenia, so both oncology and infectious disease consultant suggested not keeping the patient on chronic antibiotics, if possible. 

One week after, the patient came for a routine follow-up. Both wounds were checked and seemed to heel nicely with no sign of external irritation, normal blood labs, and negative results of the pathology tissues taken from the wound. The patient is actually followed by an infectious disease specialist and seems to be feeling well five months after the procedure. 

## Discussion

Either for pocket or lead infection, complete removal of the foreign body is advocated as the best-recommended strategy [9]. Some reports have suggested that conservative treatment can be successful with lead preservation and extensive debridement of the pocket with irrigation and antibiotics [10-16].

CIED infection is the result of an interaction between the device itself, host, and microorganism. Endothelization can occur within a week of implantation and can protect against infection. Interaction between host and microbe is mediated with several physical and chemical properties. Adherence of these organisms to the device creates a biofilm resistant to antibiotics and the host immunity system. Infection that appears years after implantation, as in this case, could be associated with several risk factors in frail patients such as diabetes, heart failure, male with age more than 60, patient on anticoagulation for intermittent atrial fibrillation, as in this case, and patient with prosthetic valve (TAVR) [17].

Historically, conservative treatment with specific systemic antibiotics, extensive debridement, irrigation, and relocation of the device was somehow successful in certain specific cases. The conservative therapy itself was associated with extensive resection of the non-viable tissues and non-essential foreign materials, appropriate irrigation with antibiotics, and careful hemostasis with mechanical and chemical sterilization with close follow up of the patient [16].

Years ago, Furman et al. [18] successfully treated a local pocket infection without any extraction. In addition, Hurst et al. [19] successfully treated a pacemaker pocket infection with local debridement and antibiotics with no signs of recurrence after three and 70 months. 

We consider these techniques as a viable option in the era of extraction in a limited group of selected frail patients, as our patient is [16]. These patients need to be frail or very sick to undergo an extraction; the infection must be a local or pocket erosion with no systemic extension, as in this case. 

As from the guidelines, an abandoned lead has to be left in a condition that will permit in the future a possible extraction and prevent any retraction into the vessel; as in this case, the abandoned leads were capped properly in case of a possible recurrence in the future [1].

So, age, medical comorbidities, and the patient's contribution to the decision-making are important in such complex cases. As in this case, a two-step technique could be recommended, and implantation of a new system in the contralateral site is suggested.

## Conclusions

Despite extraction being the gold standard of treatment in most cases of device local and systemic infection, there are some frail patients with several comorbidities where extraction is unbearable due to its major risks and complex procedure. In these specific cases with local infection and device erosion and no signs of any systemic infection, conservative therapy could be a viable option.
